# Differential diagnosis of cemento-osseous dysplasia and periapical cyst using texture analysis of CBCT

**DOI:** 10.1186/s12903-024-04208-7

**Published:** 2024-04-11

**Authors:** Sanghee Park, Su-Jin Jeon, Han-Gyeol Yeom, Min-Seock Seo

**Affiliations:** 1 Department of Conservative Dentistry, Wonkang University Daejeon Dental Hospital, 77 Dunsan-Ro, Seo-Gu, Daejeon, 302-120 Republic of Korea; 2 Department of Oral and Maxillofacial Radiology and Wonkwang Dental Research Institute, College of Dentistry, Wonkwang University, Iksan, Republic of Korea

**Keywords:** Cemento-osseous dysplasia, Periapical cyst, Texture analysis, Cone-beam computed tomography

## Abstract

**Background:**

Radiolucencies found at the root apex in patients with cemento-osseous dysplasia (COD) may be mistaken for periapical cysts (PC) of endodontic origin. The purpose of this study was to examine the utility of quantitative texture analysis using cone-beam computed tomography (CBCT) to differentiate between COD and PC.

**Methods:**

Patients who underwent CBCT at Wonkwang University Daejeon Dental Hospital between January 2019 and December 2022 and were diagnosed with COD and PC by clinical, radiologic, and, if necessary, histopathologic examination were included. Twenty-five patients each were retrospectively enrolled in the COD and PC group. All lesions observed on axial CBCT images were manually segmented using the open-access software MaZda version 4.6 to establish the regions of interest, which were then subjected to texture analysis. Among the 279 texture features obtained, 10 texture features with the highest Fisher coefficients were selected. Statistical analysis was performed using the Mann-Whitney U-test, Welch’s t-test, or Student’s t-test. Texture features that showed significant differences were subjected to receiver operating characteristics (ROC) curve analysis to evaluate the differential diagnostic ability of COD and PC.

**Results:**

The COD group consisted of 22 men and 3 women, while the PC group consisted of 14 men and 11 women, showing a significant difference between the two groups in terms of sex (*p*=0.003). The 10 selected texture features belonged to the gray level co-occurrence matrix and included the sum of average, sum of entropy, entropy, and difference of entropy. All 10 selected texture features showed statistically significant differences (*p*<0.05) when comparing patients with COD (*n*=25) versus those with PC (*n*=25), osteolytic-stage COD (*n*=11) versus PC (*n*=25), and osteolytic-stage COD (*n*=11) versus cementoblastic-stage COD (*n*=14). ROC curve analysis to determine the ability to differentiate between COD and PC showed a high area under the curve ranging from 0.96 to 0.98.

**Conclusion:**

Texture analysis of CBCT images has shown good diagnostic value in the differential diagnosis of COD and PC, which can help prevent unnecessary endodontic treatment, invasive biopsy, or surgical intervention associated with increased risk of infection.

## Background

Cemento-osseous dysplasia (COD) is the most frequent benign fibrous lesion of the jaw andis characterized by the replacement of periapical bone with fibrous tissue, including bone and cementum-like tissue. The etiology is unknown, but the lesion is assumed to be caused by periodontal ligament cell proliferation [1]. COD is categorized into three primary subtypes: periapical, focal, and florid. Notably, a fourth subtype, familial florid cemento-osseous dysplasia, was introduced in the updated classification in 2022 [2].

Periapical cysts (PC) may result from inflammation that irritates the epithelial tissue at the apex of a non-vital tooth. Teeth affected by a periapical cyst typically do not exhibit any response to pulp vitality tests, such as thermal and electrical pulp testing. A circular radiolucent area surrounds the apex of the affected tooth, and a loss of lamina dura can be observed along the adjacent root.

Radiolucency at the root apex is a radiographic characteristic of the osteolytic stage of COD. This can be mistaken for an endodontic periapical lesion. The main difference between the two is the presence or absence of pulp vitality [3]. However,a pulp vitality test may be unreliable or impossible if the tooth has already undergone endodontic treatment, presents pulp chamber restriction in elderly patients, has recently suffered trauma, or exhibits large restorations. Accurate differential diagnosis is important to avoid unnecessary root canal treatment and surgical intervention [4]. A retrospective study of biopsies reported that COD was present in 27 (0.4%) of 6704 biopsies clinically diagnosed as endodontic lesions [5].

Texture analysis is a branch of image processing that extracts texture descriptors from images and has been used to evaluate subtle pathological changes that are not easily identified by visual inspection. It has demonstrated its capability to enhancediagnostic precision and the ability to predict prognosis [6]. In dentistry, texture analysis using computed tomography (CT) scans, magnetic resonance imaging, and cone-beam computed tomography (CBCT) is currently being used for quantitative analysis of various diseases [6–8]. However, to the best of our knowledge, no previous study has quantitatively used texture analysis to assess the difference between COD and PC.

The objective of this study was to assess the diagnostic effectiveness of CBCT texture analysis in quantitatively evaluating COD and PC.

## Methods

### Ethical statement

This study was approved by the Institutional Review Board of Daejeon Dental Hospital, Wonkwang University College of Dentistry (No. W2304/006-001).

### Patient selection

The subjects in this study were selected from patients diagnosed with COD and PC based on radiographic, clinical, follow-up, and, if necessary, histopathologic data who underwent CBCT at Wonkwang Daejeon Dental Hospital between January 2019 and December 2022.

The exclusion criteria for COD patients included the following: inability to undergo pulp vitality test, signs of infection, and difficult visualization due to artifacts. Of the 40 patients diagnosed with COD by an endodontist after the aforementioned clinical and radiologic evaluations, six patients were excluded because root canal treatment had already been performed, five patients were excluded because a pulp vitality test was not possible owing to severe calcification of the pulp or prosthesis, and two patients were excluded because they had displayed signs of infection. Two patients were excluded because of the presence of artifacts, which made detailed visualization of the images difficult. The exclusion criteria allowed the selection of 25 of the 40 patients diagnosed with COD. Of the patients with COD, 11 were in the osteolytic stage, while 14 were in the cementoblastic stage.

Because all lesions in the patients with COD were located in the mandible, the same number of patients with mandibular lesions diagnosed as PC by histological examination were selected. Patients in the PC group had preoperative CBCTs taken during the same period, had teeth with no pulp vitality, and underwent a biopsy of lesions in the Daejeon Dental Hospital, Wonkwang University College of Dentistry. Twenty-five patients with the most recent clinical, radiologic, and histopathologic examinations were selected as controls.

### Image protocol

All CBCT examinations were conducted using the Green 21 system (Vatech, Hwaseong, Korea). The following CBCT scanning parameters were used: tube voltage, 106 kV; tube current, 4.5 mA; field-of-view, 17 × 15 cm; voxel size, 0.3 mm; and acquisition time, 18 s. The image was an axially reformatted image with a thickness of 1 mm. A medical liquid crystal display monitor (Barco E-3621, Munkyoeng, Korea) was used to interpret CBCT images.

### Image analysis and assessment

**Fig. 1:**
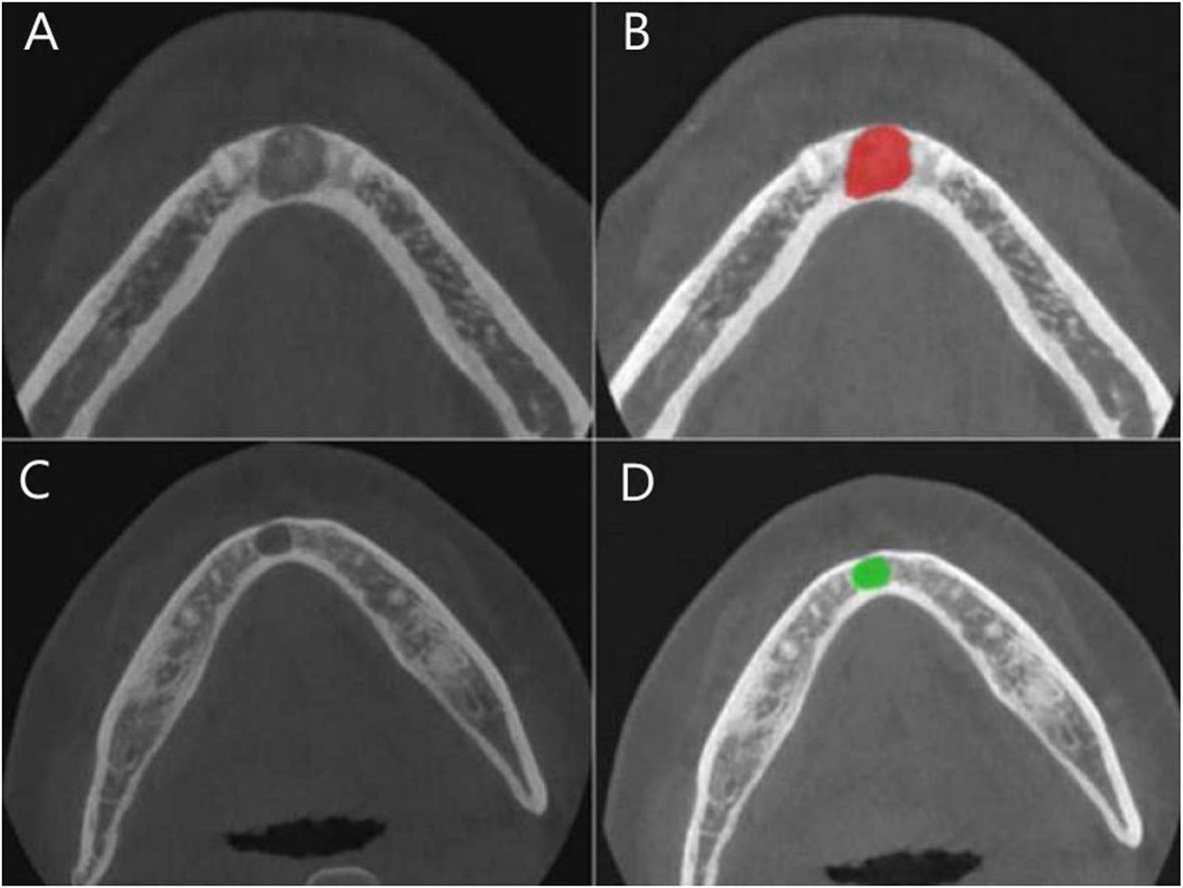
Example of region of interest placement. A 48-year-old woman with a diagnosis of cemento-osseous dysplasia (COD) (**A**). The cone-beam computed tomography (CBCT) image of the cementoblastic stage showed a radiopaque mass in the radiolucent lesion. Manually polygonal outline of the region of interest (ROI) along the outer side of the COD, which is indicated by the red area (**B**). A 52-year-old man with a diagnosis of periapical cyst (PC) (**C**). The CBCT image of the periapical cyst showed a radiolucent lesion with a radiopaque edge. Manually polygonal outline of the ROI along the outer side of the PC, which is indicated by the green area (**D**)

Image normalization was performed using the default settings of the open-access MaZda version 4.6 from the Institute of Electronics at the Technical University of Lodz in Poland [9]. An oral and maxillo-facial radiologist with 9 years of experience selected the most characteristic axial image of the COD and PC and segmented each region of interest (ROI) of the selected axial images. The COD and PC groups were defined using two distinct colors, and the ROI along the outer edge of the lesion was manually polygonal outlined (Fig. 1).

In the MaZda feature selection settings, the following image analysis techniques were selected: histogram analysis, absolute gradient, gray-level run length matrix, gray-level co-occurrence matrix (GLCM), and autoregressive model. In the bitmap image file format, 279 texture features were retrieved from each ROI on CBCT images. The Fisher method was used to determine the 10 features with the largest F coefficient among them in MaZda.

### Statistical analysis

A comparison was made between patient characteristics and texture features of patients diagnosed with COD and those with PC. Next, differences in the same texture parameters were analyzed as the difference between osteolytic-stage COD and PC and between osteolytic-stage COD and cementoblastic-stage COD. Categorical variables related to patient characteristics were examined using the Chi-square test. To assess differences between COD and PC for continuous variables based on the 10 texture features, the Student t-test, Welch’s t-test, or Mann-Whitney U test was used. The choice of test depended on the results of the Shapiro-Wilk test and F-test for continuous variables, as applicable. Wilcoxon rank-sum test was used to evaluate the difference between osteolytic-stage COD and PC and between osteolytic-stage COD and cementoblastic-stage COD using the same 10 texture features. The area under the curve (AUC) was computed following the analysis of receiver operating characteristics (ROC) curves to evaluate the predictive capacity of texture features in determining COD. The Youden index was used to establish cutoff values, and statistical significance was defined as *p*<0.05. All analyses were performed using R, version 3.6.3 (R Development Core Team in Auckland, New Zealand).

## Results

The mean age of the patients with COD was 41.28 years, while that of patients with PC was 43.64 years. COD lesions were located near the root apex of 10 mandibular anterior incisors, 7 mandibular premolars, and 8 mandibular molars, whereas PC lesions were located near the root apex of 6 mandibular anterior incisors, 6 mandibular premolars, and 13 mandibular molars. No significant differences in patient characteristics were observed between the two groups, except for sex (Table 1). Approximately 88% of patients with COD were women, showing a higher prevalence in women.

**Table 1:** Distribution of patient characteristics

**Factors**	**COD (*n*=25)**	**PC (*n*=25)**	**Chi-square test**
			***p*-value**
**Age (mean ± SD)**	41.28 ± 13.07	43.64 ± 15.51	0.563
**Sex**			
Male	3 (12.0)	14 (56.0)	NA
Female	22 (88.0)	11 (44.0)	0.003*
**Lesion location**			
Anterior	10 (40.0)	6 (24.0)	0.322
Premolar	7 (28.0)	6 (24.0)	NA
Molar	8 (32.0)	13 (52.0)	NA

*COD* cemento-osseous dysplasia, *PC* periapical cyst

^*^p<0.05

Table 2 shows the 10 texture features selected as the largest F coefficients between COD and PC. It consists of 10 GLCMs, all of which displayed statistically significant differences between COD and PC (*p*<0.05).

**Table 2:** Radiomics features differentiating between COD and PC

**Texture features** **(GLCM)**	**COD** **(*n*=25)**	**PC** **(*n*=25)**	**Mann Whitney U-test**	**Welch’s t test**	**Student t-test**
			***p*-value**		
S(4,4) Sum of Average	65.15 ± 2.96	59 ± 1.45		<0.001**	
S(1,0) Sum of Entropy	1.7 ± 0.09	1.46 ± 0.1			<0.001**
S(0,1) Sum of Entropy	1.7 ± 0.1	1.46 ± 0.09			<0.001**
S(1,0) Entropy	2.26 ± 0.18	1.86 ± 0.11		<0.001**	
S(0,1) Entropy	2.24 ± 0.19	1.81 ± 0.12		<0.001**	
S(2,0) Difference of Entropy	1.2 ± 0.08	1 ± 0.07	<0.001**		
S(2,2) Difference of Entropy	1.3 ± 0.11	1.05 ± 0.07	<0.001**		
S(3,0) Difference of Entropy	1.28 ± 0.09	1.06 ± 0.08	<0.001**		
S(3,3) Difference of Entropy	1.35 ± 0.1	1.09 ± 0.08	<0.001**		
S(4,0) Difference of Entropy	1.33 ± 0.09	1.08 ± 0.08	<0.001**		

Continuous variables in the table expressed as means ± SD

*COD* cemento-osseous dysplasia, *PC* periapical cyst, *GLCM* gray level co-occurrence matrix

^**^p<0.001

The texture analysis comparison of osteolytic-stage COD with radiolucent lesion and PC using the same 10 texture features is presented in Table 3. All GLCM features also displayed statistically significant differences (*p*<0.05). Table 4 shows the results of texture analysis of osteolytic- and cementoblastic-stage COD with mixed radiolucent and radiopaque lesions using the same 10 features. All GLCM features also showed statistically significant differences (*p*<0.05), although the differences were smaller than those in previous comparisons.

**Table 3:** Radiomics features differentiating between osteolytic-stage COD and PC

**Texture features** **(GLCM)**	**Osteolytic-stage COD (*n*=11)**	**PC (*n*=25)**	**Wilcoxon rank-sum test**
			***p*-value**
S(4,4) Sum of Average	63.49 ± 2.53	59.00 ± 1.45	<0.001**
S(1,0) Sum of Entropy	1.63 ± 0.07	1.46 ± 0.095	<0.001**
S(0,1) Sum of Entropy	1.62 ± 0.07	1.46 ± 0.094	<0.001**
S(1,0) Entropy	2.12 ± 0.15	1.86 ± 0.11	<0.001**
S(0,1) Entropy	2.10 ± 0.17	1.812 ± 0.12	<0.001**
S(2,0) Difference of Entropy	1.15 ± 0.096	1.00 ± 0.07	<0.001**
S(2,2) Difference of Entropy	1.23 ± 0.13	1.05 ± 0.075	<0.001**
S(3,0) Difference of Entropy	1.23 ± 0.097	1.06 ± 0.076	<0.001**
S(3,3) Difference of Entropy	1.29 ± 0.10	1.089 ± 0.082	<0.001**
S(4,0) Difference of Entropy	1.26 ± 0.10	1.083 ± 0.08	<0.001**

Continuous variables in the table expressed as means ± SD

*COD* cemento-osseous dysplasia, *PC* periapical cyst, *GLCM* gray level co-occurrence matrix

^**^p<0.001

**Table 4:** Radiomics features differentiating between osteolytic-stage COD and cementoblastic-stage COD

**Texture features** **(GLCM)**	**Osteolytic-stage COD (*n*=11)**	**Cementoblastic-stage COD (*n*=14)**	**Wilcoxon rank-sum test**
			***p*-value**
S(4,4) Sum of Average	63.49 ± 2.53	66.45 ± 2.66	0.011*
S(1,0) Sum of Entropy	1.63 ± 0.07	1.76 ± 0.06	<0.001**
S(0,1) Sum of Entropy	1.62 ± 0.07	1.76 ± 0.065	<0.001**
S(1,0) Entropy	2.12 ± 0.15	2.38 ± 0.11	<0.001**
S(0,1) Entropy	2.10 ± 0.17	2.36 ± 0.12	<0.001**
S(2,0) Difference of Entropy	1.15 ± 0.096	1.24 ± 0.05	0.011*
S(2,2) Difference of Entropy	1.23 ± 0.13	1.35 ± 0.05	0.009*
S(3,0) Difference of Entropy	1.23 ± 0.097	1.33 ± 0.04	0.004*
S(3,3) Difference of Entropy	1.29 ± 0.10	1.40± 0.05	<0.001**
S(4,0) Difference of Entropy	1.26 ± 0.10	1.38 ± 0.04	<0.001**

Continuous variables in the table expressed as means ± SD

*COD* cemento-osseous dysplasia, *GLCM* gray level co-occurrence matrix

^*^p<0.05

^**^p<0.001

**Fig. 2:**
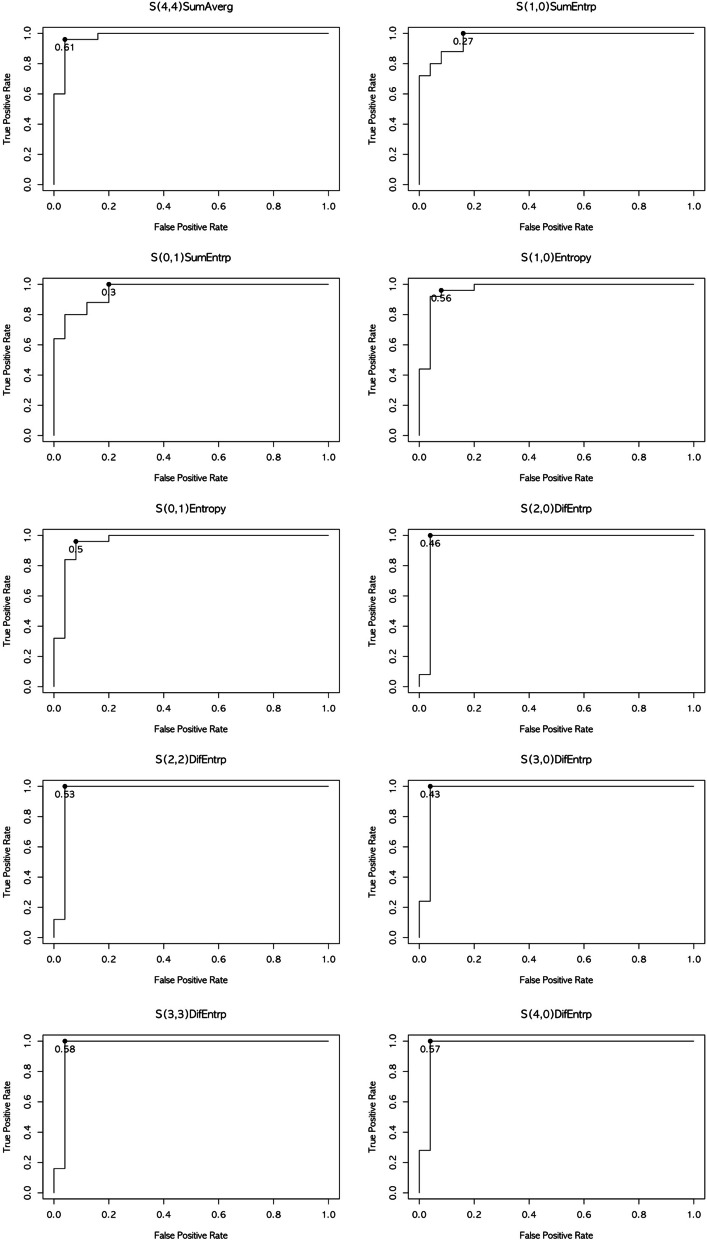
Receiver operating characteristics curves of related texture features for detecting cemento-osseous dysplasia. The graph shows the Receiver operating characteristics curves of the grey-level co-occurrence matrix for predicting cemento-osseous dysplasia. COD cemento-osseous dysplasia, SumAverg, sum of average, SumEntrp, sum of entropy, DifEntrp, difference of entropy

Figure 2 shows the ROC curves for the 10 selected texture features for detecting COD. S(4,4) sum of average, S(1,0) sum of entropy, S(0,1) sum of entropy, S(1,0) entropy, S(0,1) entropy, S(2,0) difference of entropy, S(2,2) difference of entropy, S(3,0) difference of entropy, S(3,3) difference of entropy, and S(4,0) difference of entropy had cutoff values ≥ 0.610, 0.270, 0.300, 0.560, 0.500, 0.460, 0.530, 0.430, 0.580, and 0.570, respectively.

The diagnostic performance of radiomics features for COD detection is shown in Table 5. The AUCs of 10 radiomics parameters demonstrated high accuracy between 0.88 and 0.96.

**Table 5:** Diagnostic performances of texture features to predict COD

**Texture features (GLCM)**	**Threshold criterion**	**TP**	**FP**	**FN**	**TN**	**Sensitivity (%) (95% CI)**	**Specificity (%) (95% CI)**	**Accuracy (95% CI)**	**AUC (95% CI)**
S(4,4) Sum of Average	≥0.610	23	1	2	24	0.92 (0.876, 0.947)	0.96 (0.921, 0.995)	0.94 (0.912, 0.957)	0.9792 (0.965, 0.993)
S(1,0) Sum of Entropy	≥0.270	24	4	1	21	0.96 (0.889, 0.988)	0.84 (0.775, 0.937)	0.9 (0.865, 0.93)	0.9712 (0.956, 0.986)
S(0,1) Sum of Entropy	≥0.300	24	5	1	20	0.96 (0.825, 1.00)	0.8 (0.721, 0.952)	0.88 (0.843, 0.915)	0.96 (0.94, 0.979)
S(1,0) Entropy	≥0.560	23	2	2	23	0.92 (0.851, 0.948)	0.92 (0.884, 0.997)	0.92 (0.889, 0.95)	0.9696 (0.949, 0.99)
S(0,1) Entropy	≥0.500	23	2	2	23	0.92 (0.875, 0.949)	0.92 (0.868, 0.97)	0.92 (0.886, 0.945)	0.9616 (0.937, 0.987)
S(2,0) Difference of Entropy	≥0.460	24	1	1	24	0.96 (0.944, 0.956)	0.96 (0.926, 0.994)	0.96 (0.939, 0.972)	0.9632 (0.932, 0.994)
S(2,2) Difference of Entropy	≥0.530	24	1	1	24	0.96 (0.944, 0.956)	0.96 (0.926, 0.994)	0.96 (0.939, 0.972)	0.9648 (0.935, 0.995)
S(3,0) Difference of Entropy	≥0.430	24	1	1	24	0.96 (0.944, 0.956)	0.96 (0.926, 0.994)	0.96 (0.939, 0.972)	0.9696 (0.944, 0.996)
S(3,3) Difference of Entropy	≥0.580	24	1	1	24	0.96 (0.944, 0.956)	0.96 (0.926, 0.994)	0.96 (0.939, 0.972)	0.9664 (0.938, 0.995)
S(4,0) Difference of Entropy	≥0.570	24	1	1	24	0.96 (0.944, 0.956)	0.96 (0.926, 0.994)	0.96 (0.939, 0.972)	0.9712 (0.947, 0.996)

*COD* cemento osseous dysplasia, *TP* true positive, *FP* false positive, *FN* false negative, *TN* true negative, *AUC* area under the curve, *GLCM* gray level co-occurrence matrix

## Discussion

Consistent with previous studies, patients diagnosed with COD had an average age of 41.28 ± 13.07 years and were predominantly women (88%). This finding is similar to the report that COD primarily affects Asian women, especially those in their 40s and 50s [10]. These findings support previous studies showing that hormonal factors have a complex effect on the development of the disease [11].

The findings of this study revealed a significant difference in texture analysis between COD and PC patients. Among 279 texture features, COD and PC revealed significant differences in 10 GLCM features. The 10 features included entropy, sum of average, sum of entropy, and difference of entropy.

Open-access MaZda version 4.6 offers three different analysis methods: statistical, model-based (fractal or stochastic model), and image transform (Fourier, Gabor, or wavelet transforms) [12]. The GLCM is a second-order statistical method used for texture analysis in 2D images, but it has also been extended to 3D surfaces. GLCM captures different combinations of pixel brightness values in an image and can compute features for single orientations or combine them for direction-independent analysis. However, a significant drawback is the computational cost, which can be addressed by combining GLCM with the Sobel operator to reduce processing time [13].

Specific orientations or directions are considered when computing GLCM features. Texture feature names include four directions and a distance from one to five. For example, we would represent a distance of two in a horizontal direction as (2,0), and a distance of two in a vertical direction as (0,2) [12]. Both entropy (the degree of disorder between pixels in an image) and sum of entropy (the disorganization of the sum distribution of gray shades) increased in CBCT images of patients with COD. This is thought to be due to a mixture of fibrous stroma with loose fibroblasts and collagen with mineralized tissue in COD. Large differences between adjacent pixels increase non-uniformities, and thus the sum of average (a mean of the distribution of the sum of gray shades) increases in CBCT images of patients with COD. In the CBCT images of patients with COD, the difference of entropy, which represents a disorganization of the gray shade difference, increased. This is because the periapical bone of COD patients is replaced by fibrous tissue, including bone and cementum like tissue, resulting in different shades of gray for each tissue in the CBCT image.

Although the sample size was very small, texture analysis showed a statistically significant difference between osteolytic-stage COD and PC, suggesting that the qualitative radiologic assessment was similar but the quantitative assessment was different between the two. The 10 selected variables showed a greater difference between PC and osteolytic-stage COD than the difference between the two COD stages, suggesting that the variables are diagnostically valuable indicators to differentiate COD from PC using texture analysis. This difference is thought to be due to differences in the histologic composition within the lesions. COD has fibrous tissue containing histologically woven bone or cementum-like tissue, whereas PC contains semi-solid necrotic material and cellular components due to liquefaction of epithelial cells [1, 14]. Since the 10 texture features were selected to distinguish between CODs and PCs, we believe that the results would be different if the texture features were selected to distinguish between osteolytic-stage and cementoblastic-stage CODs.

Depending on the stage of development of the lesion, CODs exhibit different radiologic characteristics [15, 16]. Qualitative assessment of CBCT findings by human eyes is possible between COD and PC. However, as noted in many case reports, misdiagnoses often occur, and qualitative assessments that rely solely on the human eye can be unreliable [3, 17, 18]. In fact, owing to the difficulty in differential diagnosis of the two, 7 of the 25 patients diagnosed with COD in this study had to undergo biopsy for confirmatory diagnosis. MDCT is believed to be more accurate than CBCT at very osteolytic stages; however, it is not the first choice because of the high radiation dose [19]. Therefore, texture analysis using CBCT is a non-invasive method that can compensate for these shortcomings, provide a quantitative assessment, and prevent endodontic or surgical intervention.

The ROC analysis of this study yielded 10 cutoff values for differentiating PC and COD, calculated by the Youden index. It also provided a high level of accuracy, ranging from 0.88 to 0.96. This indicates that CBCT texture analysis helps differentiate between COD and PC. Therefore, the analysis of CBCT texture features can provide a quantitative assessment of COD to prevent unnecessary endodontic or surgical intervention, thereby reducing the risk of infection from biopsy.

CBCT evaluation has recently increased and is primarily used for COD diagnosis, as it allows three-dimensional imaging with minimal distortion, high spatial resolution, and no overlap [20]. A comprehensive evaluation of demographic, clinical, radiologic, and follow-up information is typically used to diagnose COD [11, 21]. A biopsy is contraindicated in these lesions because of the deposition of cementum-like tissue and reduced local vascularity, which increases the risk of infection [10, 11, 15, 22]. Generally, COD does not require treatment because routine radiological follow-up is the preferred therapeutic modality [23]. Therefore, many previous studies have used CBCT rather than invasive histologic biopsy to qualitatively diagnose COD [24, 25]. The diagnosis of COD, which was previously based on a qualitative evaluation using CBCT, can be quantitatively assessed using texture analysis as a new non-invasive method to further differentiate it from PC.

Owing to the large number of scattered rays that enter the detector, CBCT is typically not appropriate for calculating quantitative texture features [26]. Even if the CBCT scan time is shorter than the CT scan time, the texture parameters can be affected by motion artifacts. However, some studies using CBCT texture analysis for maxillofacial diseases have been published recently [8, 27, 28]. De Rosa et al. performed CBCT texture analysis to distinguish between a radicular cyst and a periapical granuloma [27]. Goncalves et al. performed CBCT texture analysis to detect furcal lesions [8]. Cost et al. performed CBCT texture analysis to analyze alveolar bone features to confirm implant stability in the maxillary edentulous area [28]. In addition, according to earlier research, some CBCT radiomics features can be used as quantitative indicators for texture analysis [29].

A limitation of this study is that texture analysis was conducted using a small sample size. This is because the lesions are usually asymptomatic and are often discovered by chance on routine dental radiographs performed for other reasons. Future studies should evaluate osteolytic-stage COD using a larger sample size.

## Conclusions

This study demonstrated quantitative differences between COD and PC using noninvasive CBCT texture analysis. Texture features can be used as a quantitative indicator for diagnosing COD and may help prevent endodontic treatment, invasive biopsy, or surgical intervention, which are associated with an increased risk of infection.

## Data Availability

The authors have full control of all primary data and have agreed to allow the journal to review the data if requested. The datasets used and/or analyzed during the current study are available from the corresponding author on reasonable request. The experimental data that support the findings of this study are available in Figshare with the identifier URL: 10.6084/m9.figshare.25377631.

## Abbreviations

COD: cemento-osseous dysplasia
PC: periapical cyst
CBCT: cone-beam computed tomography
ROI: region of interest
ROC: receiver operating characteristic
GLCM: gray level co-occurrence matrix
AUC: area under the curve
